# Supplementary material for: Structure-based pharmacophore modeling, high-throughput screening, and molecular dynamics identify a novel DrugBank-derived HIV-1 protease inhibitor
Source: PLoS One. 2026 Jun 12;21(6):e0336765. doi: 10.1371/journal.pone.0336765 (PMC13262882; doi:10.1371/journal.pone.0336765)
Supplement: Attachment — Submitted filename: comments.docx [file pone.0336765.s003.docx]

1. Introduce the abbreviation “BAN” for the ligand of interest in the abstract
2. Is convergence based on just one production? I suggest running independent experiments for each system or inhibitor to ensure 3TL's performance isn't merely due to limited sampling. From the replicates uncertainties and convergence test can be plausible. This will avoid over interpretation of small differences between your systems.
3. The reported SEM of 0 in (see Table 1) is not realistic for molecular dynamics data. MD trajectories are inherently noisy and time-correlated, so some level of uncertainty is always expected. This suggests that the SEM may have been calculated incorrectly or without accounting for autocorrelation. The authors should clarify their methodology and apply an appropriate error estimation approach (e.g., block averaging).
4. The manuscript contains numerous typographical and grammatical errors throughout the text. Careful proofreading and language editing are required to improve clarity and readability.
