# Supplementary material for: Structure-based pharmacophore modeling, high-throughput screening, and molecular dynamics identify a novel DrugBank-derived HIV-1 protease inhibitor
Source: PLoS One. 2026 Jun 12;21(6):e0336765. doi: 10.1371/journal.pone.0336765 (PMC13262882; doi:10.1371/journal.pone.0336765)
Supplement: Attachment — Submitted filename: Response to Reviewers 01.docx [file pone.0336765.s004.docx]

April 23, 2026

Dear Editorial of PLOS ONE

Response to Editorial Requests

1. We thank the editorial office for the opportunity to update our submission. We have now provided validated ORCID ID, as requested.

2. Regarding the Data Availability Statement, we confirm that our submission does contain all data required to replicate the study findings. The 'minimal data set' is contained within the manuscript tables and the Supporting Information Table S1 file. Therefore, we confirm that our original statement, is accurate and the work can be fully replicate. We have reviewed the revised PDF and are pleased to resubmit our manuscript for consideration.

Again, we are appreciating the time, consideration, that helped us to improve the manuscript in different aspects.

Bests

Amjed Majid Rashid Alrashedi

Department of Biochemistry, Faculty of Science, Kashan State University, Kashan, Iran

Email: [amjad93majid11@gmail.com](mailto:amjad93majid11@gmail.com)
