# Supplementary material for: Structure-based pharmacophore modeling, high-throughput screening, and molecular dynamics identify a novel DrugBank-derived HIV-1 protease inhibitor
Source: PLoS One. 2026 Jun 12;21(6):e0336765. doi: 10.1371/journal.pone.0336765 (PMC13262882; doi:10.1371/journal.pone.0336765)
Supplement: Peer review history

# Peer review: Decision Letter 0

**DOI:** 10.1371/journal.pone.0336765.r001

**Authors:** Soumendranath Bhakat

9 Mar 2026

PONE-D-25-58713

Structure-Based Pharmacophore Modeling, High-throughput Screening, and Molecular Dynamics Identify a Novel DrugBank-Derived HIV-1 Protease Inhibitor

PLOS One

Dear Dr. Rashid Alrashedi,

Thank you for submitting your manuscript to PLOS ONE. After careful consideration, we feel that it has merit but does not fully meet PLOS ONE’s publication criteria as it currently stands. Therefore, we invite you to submit a revised version of the manuscript that addresses the points raised during the review process.

==============================

**Editor's comment:**

**In view of the reviewer’s request for further statistical analysis, I invite you to revise and resubmit your manuscript. I look forward to reviewing the updated version.**

==============================

Please submit your revised manuscript by Apr 23 2026 11:59PM. If you will need more time than this to complete your revisions, please reply to this message or contact the journal office at plosone@plos.org. When you're ready to submit your revision, log on to https://www.editorialmanager.com/pone/ and select the 'Submissions Needing Revision' folder to locate your manuscript file.

Please include the following items when submitting your revised manuscript:

- A letter that responds to each point raised by the academic editor and reviewer(s). You should upload this letter as a separate file labeled 'Response to Reviewers'.
- A marked-up copy of your manuscript that highlights changes made to the original version. You should upload this as a separate file labeled 'Revised Manuscript with Track Changes'.
- An unmarked version of your revised paper without tracked changes. You should upload this as a separate file labeled 'Manuscript'.

-->If you would like to make changes to your financial disclosure, please include your updated statement in your cover letter. Guidelines for resubmitting your figure files are available below the reviewer comments at the end of this letter.

If applicable, we recommend that you deposit your laboratory protocols in protocols.io to enhance the reproducibility of your results. Protocols.io assigns your protocol its own identifier (DOI) so that it can be cited independently in the future. For instructions see: https://journals.plos.org/plosone/s/submission-guidelines#loc-laboratory-protocols. Additionally, PLOS ONE offers an option for publishing peer-reviewed Lab Protocol articles, which describe protocols hosted on protocols.io. Read more information on sharing protocols at https://plos.org/protocols?utm_medium=editorial-email&utm_source=authorletters&utm_campaign=protocols.

We look forward to receiving your revised manuscript.

Kind regards,

Soumendranath Bhakat

Academic Editor

PLOS One

Journal Requirements:

When submitting your revision, we need you to address these additional requirements.

1. Please ensure that your manuscript meets PLOS ONE's style requirements, including those for file naming. The PLOS ONE style templates can be found at

https://journals.plos.org/plosone/s/file?id=wjVg/PLOSOne_formatting_sample_main_body.pdf and https://journals.plos.org/plosone/s/file?id=ba62/PLOSOne_formatting_sample_title_authors_affiliations.pdf

2. PLOS requires an ORCID iD for the corresponding author in Editorial Manager on papers submitted after December 6th, 2016. Please ensure that you have an ORCID iD and that it is validated in Editorial Manager. To do this, go to ‘Update my Information’ (in the upper left-hand corner of the main menu), and click on the Fetch/Validate link next to the ORCID field. This will take you to the ORCID site and allow you to create a new iD or authenticate a pre-existing iD in Editorial Manager.

3. Please include a complete copy of PLOS’ questionnaire on inclusivity in global research in your revised manuscript. Our policy for research in this area aims to improve transparency in the reporting of research performed outside of researchers’ own country or community. The policy applies to researchers who have travelled to a different country to conduct research, research with Indigenous populations or their lands, and research on cultural artefacts. The questionnaire can also be requested at the journal’s discretion for any other submissions, even if these conditions are not met.  Please find more information on the policy and a link to download a blank copy of the questionnaire here: https://journals.plos.org/plosone/s/best-practices-in-research-reporting. Please upload a completed version of your questionnaire as Supporting Information when you resubmit your manuscript.

4. Please note that PLOS One has specific guidelines on code sharing for submissions in which author-generated code underpins the findings in the manuscript. In these cases, we expect all author-generated code to be made available without restrictions upon publication of the work. Please review our guidelines at https://journals.plos.org/plosone/s/materials-and-software-sharing#loc-sharing-code and ensure that your code is shared in a way that follows best practice and facilitates reproducibility and reuse.

5. We note that your Data Availability Statement is currently as follows: All relevant data are within the manuscript and its Supporting Information files.

Please confirm at this time whether or not your submission contains all raw data required to replicate the results of your study. Authors must share the “minimal data set” for their submission. PLOS defines the minimal data set to consist of the data required to replicate all study findings reported in the article, as well as related metadata and methods (https://journals.plos.org/plosone/s/data-availability#loc-minimal-data-set-definition).

For example, authors should submit the following data:

- The values behind the means, standard deviations and other measures reported;

- The values used to build graphs;

- The points extracted from images for analysis.

Authors do not need to submit their entire data set if only a portion of the data was used in the reported study.

If your submission does not contain these data, please either upload them as Supporting Information files or deposit them to a stable, public repository and provide us with the relevant URLs, DOIs, or accession numbers. For a list of recommended repositories, please see https://journals.plos.org/plosone/s/recommended-repositories.

If there are ethical or legal restrictions on sharing a de-identified data set, please explain them in detail (e.g., data contain potentially sensitive information, data are owned by a third-party organization, etc.) and who has imposed them (e.g., an ethics committee). Please also provide contact information for a data access committee, ethics committee, or other institutional body to which data requests may be sent. If data are owned by a third party, please indicate how others may request data access.

6. If the reviewer comments include a recommendation to cite specific previously published works, please review and evaluate these publications to determine whether they are relevant and should be cited. There is no requirement to cite these works unless the editor has indicated otherwise.

7. Please review your reference list to ensure that it is complete and correct. If you have cited papers that have been retracted, please include the rationale for doing so in the manuscript text, or remove these references and replace them with relevant current references. Any changes to the reference list should be mentioned in the rebuttal letter that accompanies your revised manuscript. If you need to cite a retracted article, indicate the article’s retracted status in the References list and also include a citation and full reference for the retraction notice.

Additional Editor Comments ;

In view of the reviewer’s request for further statistical analysis, I invite you to revise and resubmit your manuscript. I look forward to reviewing the updated version.

[Note: HTML markup is below. Please do not edit.]

Reviewers' comments:

Reviewer's Responses to Questions

**Comments to the Author**

1. Is the manuscript technically sound, and do the data support the conclusions?

The manuscript must describe a technically sound piece of scientific research with data that supports the conclusions. Experiments must have been conducted rigorously, with appropriate controls, replication, and sample sizes. The conclusions must be drawn appropriately based on the data presented.

Reviewer #1: Partly

**********

2. Has the statistical analysis been performed appropriately and rigorously?

Reviewer #1: No

**********

3. Have the authors made all data underlying the findings in their manuscript fully available?

The PLOS Data policy requires authors to make all data underlying the findings described in their manuscript fully available without restriction, with rare exception (please refer to the Data Availability Statement in the manuscript PDF file). The data should be provided as part of the manuscript or its supporting information, or deposited to a public repository. For example, in addition to summary statistics, the data points behind means, medians and variance measures should be available. If there are restrictions on publicly sharing data—e.g. participant privacy or use of data from a third party—those must be specified.

Reviewer #1: Yes

**********

4. Is the manuscript presented in an intelligible fashion and written in standard English?

PLOS ONE does not copyedit accepted manuscripts, so the language in submitted articles must be clear, correct, and unambiguous. Any typographical or grammatical errors should be corrected at revision, so please note any specific errors here.

Reviewer #1: No

**********

5. Review Comments to the Author

Please use the space provided to explain your answers to the questions above. You may also include additional comments for the author, including concerns about dual publication, research ethics, or publication ethics. (Please upload your review as an attachment if it exceeds 20,000 characters)

Reviewer #1: The manuscript does not provide clear evidence that the reported observable has converged. Without time-dependent analysis (e.g., running averages or block comparisons), it is difficult to determine whether the observed differences reflects a stable effect or insufficient sampling.

**********

6. PLOS authors have the option to publish the peer review history of their article (what does this mean?). If published, this will include your full peer review and any attached files.

If you choose “no”, your identity will remain anonymous but your review may still be made public.

**Do you want your identity to be public for this peer review?** For information about this choice, including consent withdrawal, please see our Privacy Policy.

Reviewer #1: No

**********

[NOTE: If reviewer comments were submitted as an attachment file, they will be attached to this email and accessible via the submission site. Please log into your account, locate the manuscript record, and check for the action link "View Attachments". If this link does not appear, there are no attachment files.]

To ensure your figures meet our technical requirements, please review our figure guidelines: https://journals.plos.org/plosone/s/figures

You may also use PLOS’s free figure tool, NAAS, to help you prepare publication quality figures: https://journals.plos.org/plosone/s/figures#loc-tools-for-figure-preparation.

NAAS will assess whether your figures meet our technical requirements by comparing each figure against our figure specifications.

Attachment Submitted filename: comments.docx

# Author comment: Author response to Decision Letter 1

**DOI:** 10.1371/journal.pone.0336765.r002

23 Apr 2026

April 18, 2026

Dear Editorials of PLOS One

Thank you for the opportunity to revise and resubmit our manuscript (ID: PONE-D-25-58713) entitled ‘Structure-Based Pharmacophore Modeling, High-throughput Screening, and Molecular Dynamics Identify a Novel DrugBank-Derived HIV-1 Protease Inhibitor’. We appreciate your time, and consideration, and thoughtful comments and valuable suggestions provided by the reviewers and the editorial team. We have carefully addressed all of the concerns raised. The point-by-point changes are as follow:

In response to the Editorial comments:

Dear Editorials,

Authors’ action:

1. The global research questionnaire is filled and is uploaded as ‘S2 Checklist’ file, and referred to in the Supporting information within the revised manuscript.

2. A new section ‘Inclusivity in global research’ is included in the Methods section as recommended.

3. Statistical analysis is provided in the Methods section under the sub-heading ‘Statistical analysis’. Accordingly, the statistical findings are provided in detail in Supporting information ‘Table S1’.

4. The manuscript revised as recommended.

Reviewer’s comment:

1. Introduce the abbreviation “BAN” for the ligand of interest in the abstract

Authors’ action: Thank you for noting this oversight. The abstract has been revised to introduce the full ligand name at first mention, followed by the abbreviation “BAN” in parentheses. All subsequent references now use the abbreviation consistently.

2. Is convergence based on just one production? I suggest running independent experiments for each system or inhibitor to ensure 3TL's performance isn't merely due to limited sampling. From the replicates uncertainties and convergence test can be plausible. This will avoid over interpretation of small differences between your systems.

Authors’ action: We appreciate the suggestion to verify convergence using multiple production runs. In the revised manuscript, we have addressed a new subheading in the Methods section ‘Convergence evaluation’ on how we have determined the convergence of systems based-on production phase of the simulation for reproducibility.

3. The reported SEM of 0 in (see Table 1) is not realistic for molecular dynamics data. MD trajectories are inherently noisy and time-correlated, so some level of uncertainty is always expected. This suggests that the SEM may have been calculated incorrectly or without accounting for autocorrelation. The authors should clarify their methodology and apply an appropriate error estimation approach (e.g., block averaging).

Authors’ action: Thank you so much. Yes, the SEM findings were low and it was the result of the analysis. It is not actual ‘zero’ we have rounded all the values with three decimals, and it is consistent through the manuscript. For demonstrating the values, we have increased the rounded number for more detail in the Table S1. Further statistical analyzes are also presented in the same file.

4. The manuscript contains numerous typographical and grammatical errors throughout the text. Careful proofreading and language editing are required to improve clarity and readability.

Authors’ action: Thank you so much for this comment. The entire manuscript has undergone comprehensive proofreading and language editing. Grammar, clarity, and flow have been improved throughout the text, with corrections to punctuation, verb tenses, subject‑verb agreement, hyphenation, and scientific phrasing. These revisions enhance readability without altering scientific content.

Again, we are appreciating the time, consideration, and the editorials and dear reviewers’ comments that helped us to improve the manuscript in different aspects. I hope the revised manuscript met the journals and reviewers’ standards.

Bests

Amjed Majid Rashid Alrashedi

Department of Biochemistry, Faculty of Science, Kashan State University, Kashan, Iran

Email: amjad93majid11@gmail.com

April 23, 2026

Dear Editorial of PLOS ONE

Response to Editorial Requests

1. We thank the editorial office for the opportunity to update our submission. We have now provided validated ORCID ID, as requested.

2. Regarding the Data Availability Statement, we confirm that our submission does contain all data required to replicate the study findings. The 'minimal data set' is contained within the manuscript tables and the Supporting Information Table S1 file. Therefore, we confirm that our original statement, is accurate and the work can be fully replicate. We have reviewed the revised PDF and are pleased to resubmit our manuscript for consideration.

Again, we are appreciating the time, consideration, that helped us to improve the manuscript in different aspects.

Bests

Amjed Majid Rashid Alrashedi

Department of Biochemistry, Faculty of Science, Kashan State University, Kashan, Iran

Email: amjad93majid11@gmail.com

Attachment Submitted filename: Response to Reviewers 01.docx

# Peer review: Decision Letter 1

**DOI:** 10.1371/journal.pone.0336765.r003

**Authors:** Soumendranath Bhakat

27 May 2026

Structure-Based Pharmacophore Modeling, High-throughput Screening, and Molecular Dynamics Identify a Novel DrugBank-Derived HIV-1 Protease Inhibitor

PONE-D-25-58713R1

Dear Dr. Alrashedi,

We’re pleased to inform you that your manuscript has been judged scientifically suitable for publication and will be formally accepted for publication once it meets all outstanding technical requirements.

Within one week, you’ll receive an e-mail detailing the required amendments. When these have been addressed, you’ll receive a formal acceptance letter and your manuscript will be scheduled for publication.

An invoice will be generated when your article is formally accepted. Please note, if your institution has a publishing partnership with PLOS and your article meets the relevant criteria, all or part of your publication costs will be covered. Please make sure your user information is up-to-date by logging into Editorial Manager at Editorial Manager® and clicking the ‘Update My Information' link at the top of the page. For questions related to billing, please contact billing support.

If your institution or institutions have a press office, please notify them about your upcoming paper to help maximize its impact. If they’ll be preparing press materials, please inform our press team as soon as possible -- no later than 48 hours after receiving the formal acceptance. Your manuscript will remain under strict press embargo until 2 pm Eastern Time on the date of publication. For more information, please contact onepress@plos.org.

Kind regards,

Soumendranath Bhakat

Academic Editor

PLOS One

Additional Editor Comments (optional):

Reviewers' comments:

Reviewer's Responses to Questions

**Comments to the Author**

1. If the authors have adequately addressed your comments raised in a previous round of review and you feel that this manuscript is now acceptable for publication, you may indicate that here to bypass the “Comments to the Author” section, enter your conflict of interest statement in the “Confidential to Editor” section, and submit your "Accept" recommendation.

Reviewer #1: All comments have been addressed

**********

2. Is the manuscript technically sound, and do the data support the conclusions?

The manuscript must describe a technically sound piece of scientific research with data that supports the conclusions. Experiments must have been conducted rigorously, with appropriate controls, replication, and sample sizes. The conclusions must be drawn appropriately based on the data presented.

Reviewer #1: Yes

**********

3. Has the statistical analysis been performed appropriately and rigorously?

Reviewer #1: Yes

**********

4. Have the authors made all data underlying the findings in their manuscript fully available?

The PLOS Data policy requires authors to make all data underlying the findings described in their manuscript fully available without restriction, with rare exception (please refer to the Data Availability Statement in the manuscript PDF file). The data should be provided as part of the manuscript or its supporting information, or deposited to a public repository. For example, in addition to summary statistics, the data points behind means, medians and variance measures should be available. If there are restrictions on publicly sharing data—e.g. participant privacy or use of data from a third party—those must be specified.

Reviewer #1: Yes

**********

5. Is the manuscript presented in an intelligible fashion and written in standard English?

PLOS ONE does not copyedit accepted manuscripts, so the language in submitted articles must be clear, correct, and unambiguous. Any typographical or grammatical errors should be corrected at revision, so please note any specific errors here.

Reviewer #1: Yes

**********

6. Review Comments to the Author

Please use the space provided to explain your answers to the questions above. You may also include additional comments for the author, including concerns about dual publication, research ethics, or publication ethics. (Please upload your review as an attachment if it exceeds 20,000 characters)

Reviewer #1: (No Response)

**********

7. PLOS authors have the option to publish the peer review history of their article (what does this mean?). If published, this will include your full peer review and any attached files.

If you choose “no”, your identity will remain anonymous but your review may still be made public.

**Do you want your identity to be public for this peer review?** For information about this choice, including consent withdrawal, please see our Privacy Policy.

Reviewer #1: No

**********

# Editor report: Acceptance letter

**DOI:** 10.1371/journal.pone.0336765.r004

**Authors:** Soumendranath Bhakat

PONE-D-25-58713R1

PLOS One

Dear Dr. Rashid Alrashedi,

I'm pleased to inform you that your manuscript has been deemed suitable for publication in PLOS One. Congratulations! Your manuscript is now being handed over to our production team.

At this stage, our production department will prepare your paper for publication. This includes ensuring the following:

* All references, tables, and figures are properly cited

* All relevant supporting information is included in the manuscript submission,

* There are no issues that prevent the paper from being properly typeset

You will receive further instructions from the production team, including instructions on how to review your proof when it is ready. Please keep in mind that we are working through a large volume of accepted articles, so please give us a few days to review your paper and let you know the next and final steps.

Lastly, if your institution or institutions have a press office, please let them know about your upcoming paper now to help maximize its impact. If they'll be preparing press materials, please inform our press team within the next 48 hours. Your manuscript will remain under strict press embargo until 2 pm Eastern Time on the date of publication. For more information, please contact onepress@plos.org.

You will receive an invoice from PLOS for your publication fee after your manuscript has reached the completed accept phase. If you receive an email requesting payment before acceptance or for any other service, this may be a phishing scheme. Learn how to identify phishing emails and protect your accounts at https://explore.plos.org/phishing.

If we can help with anything else, please email us at customercare@plos.org.

Thank you for submitting your work to PLOS ONE and supporting open access.

Kind regards,

PLOS ONE Editorial Office Staff

on behalf of

Dr. Soumendranath Bhakat

Academic Editor

PLOS One
